# News and Miscellany

**Published:** 1901-08

**Authors:** 


### News and Miscellany.

---

**Dr. Russ, Jr.** Born to Doctor and Mrs. Witten Booth Russ, San Antonio, Texas, June 22, 1901, a son. Mrs. Russ was Miss Jean McGrath, of Philadelphia.

---

**Dr. J. D. Fennell,** of Seguin, Texas, died in that city June 28, 1901.

---

**The New Medical Law of Texas,** in pamphlet form, can be had by sending 20 cents to the TEXAS MEDICAL JOURNAL, Austin, Texas.

---

**Quite Feminine.**—A woman whose doctor asked after her health replied dolefully: "I feel very well; but I always feel bad when I feel well, because I know I'm going to feel worse afterward."

---

**For Sale.**—Property and practice. Practice unopposed for 40 miles, about $3000. Property, 9-room house, wind mill, good orchard, 2 barns, good repair, price $1000, terms to suit. Address, GEO. L. JACKSON, M. D., Leakey, Texas.

---

**A $3,000.00 practice,** established six years, can be had by buying $1,000 residence. No competition. Want to make climatic change.

T. L. TREADAWAY, M. D.,
Lilac, Texas.

---

**New Orleans Polyclinic.**—Physicians will find the Polyclinic an excellent means for posting themselves upon modern progress in all branches of medicine and surgery. The special ties are fully taught, particulary laboratory work. Fourteenth annual session opens November 12, 1900. For further information address Dr. Isadore Dyer, Secretary, New Orleans Polyclinic, New Orleans.

---

**The State Board of Medical Examiners,** appointed by the Governor under the new Medical Practice Law, which went into effect July 8th (ult.), met in Austin July 23rd, and organized. Dr. J. T. Wilson, of Sherman, was elected President; Dr. S. R. Burroughs, of Buffalo, Vice-President, and Dr. M. M. Smith, of Austin, Secretary and Treasurer. They formulated plans for future action, and will hold their first meeting for examining applicants, at Austin, the first Tuesday in October, it being the 1st day. Meantime the Secretary will have all the blanks and books made, and get ready. Notice of the meeting will be given through the daily papers, as well as through the medical press of the State.

---

**Dr. W. W. Walker** died at his home in Schulenburg, Texas, May 5, 1901. He was born in Louisiana in 1844. He served in the 3rd Louisiana Cavalry during the civil war, four years and nineteen days. He was wounded at second Manassas battle. Settled in Texas after the war. Studied medicine, took his degree from Tulane University Medical School in 1871. He was twice married. In 1898 Dr. Walker raised a company and entlisted it for the Cuban war, and as its captain went out in the 1st Texas Cavalry. At Santiago de Cuba he was made surgeon with the rank of captain. Dr. Walker has two sons who are physicians, Dr. E. R. Walker, of Schulenburg, and Dr. W. H. Walker, of Oakland, Texas.

---

Green is flowery tamarack;
The aspen's ghost leaves quiver;
In the right hypochondriac
Still sleeps the torpid liver.

The wind is light, the world is young;
High leaps the rutting goat;
Upon the back part of the tongue,
Appears a furry coat.

Beneath the ground are bursting seed;
In trees the birds are billin';
While men and women seem to need
A dose of podophyllin.

—*Atlanta Journal-Record of Medicine.*

### The Etiology of Alopecia.

Dr. Delos L. Parker, in the July 13th number of the *New York Medical Record,* advances an interesting theory to explain the etiology of alopecia. He begins his article by reviewing at length the anatomy of the thorax and the physiology of respiration. Briefly, his theory is as follows: Respired air contains a certain amount of organic waste matter, and when air that has become impregnated with this waste material remains in unused parts of the lung decomposition follows with the production of a certain poisonous product which has a selective action on hair follicles. This he calls hair poison or tricotoxicon. Women employ the costal type of breathing, hence there is a free interchange of air in the lungs and no hair poison is elaborated. On the other hand, men use the lower costal and abdominal types of breathing, and as a result respired air is retained in the apices of the lungs and decomposition of and absorption follow. Hence the greater frequency of alopecia in young men than in young women. In old age in both sexes difficulty in filling the lungs is increased owing to senile changes and alopecia results from the elaboration of a large amount of poison. The frequency of alopecia in individuals who lead sedentary lives is explained by the fact that they rarely have opportunity or inclination to properly expand their lungs. In consumptives alopecia is comparatively rare because the apices of their lungs are usually consolidated, or, at any rate, so diseased that their absorptive power is nil. Dr. Parker has tested his theory by injecting distilled water impregnated with decomposed expired air into dogs, hens, and pigeons. In every instance the animal or bird has lost large areas of hair or feathers, as the case might be. To prove that manipulation and interference with the animal's general health cannot account for the loss of hair thus obtained, Dr. Parker conducted a series of control experiments, using pure distilled water for the injections. The results from these were negative.

In conclusion, Dr. Parker argues that since tricotoxicon, which has the power of producing alopecia in lower animals, can be obtained by the decomposition of respired air in the presence of warmth and moisture outside of the human body, it is fair to suppose that it is elaborated within the lungs under similar conditions, and that it has a similar effect on the hair follicles of the human body. In his final paragraph he says: "From a consideration of what has been written above, it seems to the writer not unreasonable to conclude that alopecia, of the type under consideration, is caused by an auto-infection in which tricotoxicon is taken up by the blood from the air cells of the lungs, where it has been elaborated during decomposition of organic matter normally present in respired air.

